# Vocal teacher production condition in differents functional situations

**DOI:** 10.1590/2317-1782/20202020208

**Published:** 2021-12-08

**Authors:** Aline Ferreira de Brito Mota, Ariane Damasceno Pellicani, Rodrigo Dornelas, Lilian Neto Aguiar Ricz

**Affiliations:** 1 Departamento de Fonoaudiologia, Universidade Federal de Sergipe – UFS - Lagarto (SE), Brasil.; 2 Programa de Pós-graduação do Departamento de Otorrinolaringologia, Oftalmoologia e Cabeça e Pescoço, Faculdade de Medicina de Ribeirão Preto – FMRP, Universidade de São Paulo – USP - Ribeirão Preto (SP), Brasil.; 3 Departamento de Fonoaudiologia, Universidade Federal do Rio de Janeiro – UFRJ - Rio de Janeiro (RJ), Brasil.; 4 Departamento de Oftalmologia, Otorrinolaringologia e Cirurgia de Cabeça e Pescoço, Faculdade de Medicina de Ribeirão Preto – FMRP, Universidade de São Paulo – USP - Ribeirão Preto (SP), Brasil.

**Keywords:** Teachers, Dysphonia, Education, Voice, Speech, Language and Hearing Sciences

## Abstract

**Purpose:**

to verify if teachers with less vocal use due to reduced workload have fewer complaints of vocal disorders and better environmental and organizational working conditions.

**Methods:**

46 teachers of both genders, with a mean age of 39.5 years old, and 15 years of career length participated in this study. The individuals were divided into group A, public school teachers with exclusive dedication to a single school and regulated workload; group B, public school teachers with elevated workload working in many schools. All subjects were submitted to the following instruments: Condition of Vocal Production-Teacher and the Screening Index for Voice Disorder.

**Results:**

group B teachers presented voice disorder (5.21; p=0.02) and greater complaints regarding acoustic conditions (p=0.04), temperature (p=0,04), humidity (p=0.01), lighting (p=0.001), cleanliness (p=0.01), and didactic materials (p<0.0001). Habits of screaming (p=0.02), speaking in an open place (p=0,02), and vocal orientations (p=0.01) also had a statistically significant difference.

**Conclusion:**

Teachers working in elementary and high school belonging to the group of exclusive dedication to a single school, with reduced weekly classroom hours and less vocal exposure had fewer complaints of voice disorders, better environmental and organizational conditions, and reported screaming less at work.

## INTRODUCTION

Teachers are part of the professionals whose communication is a vital element for the feasibility of their work, and the voice is the instrument used to establish direct links with the student, the family, and the community^(1,2)^.

Vocal disorders may be more frequent among the population that needs to use their voice professionally, due to the high vocal demand and exposure to various risk factors. The professional voice was conceptualized as a form of oral communication, used by individuals who depend on it to carry out their occupational activity and, through this mode of expression, reach a specific and determined audience^(3,4)^.

Teachers represent the category of professionals most affected by voice disorder^(5)^. Voice disorder is defined as any change in the voice resulting from a functional and/or organic disorder of the vocal tract that makes natural voice production impossible^(6)^.

In the search for the treatment of voice disorder, teachers report that the illness has a close relationship with the environment and the organization of work. The association of teaching work conditions with impaired voice production is highlighted regarding the environmental aspects of the school (such as the noise that requires the use of the voice at a higher intensity, or the dust that triggers allergic reactions), and the organization of the teaching work (such as long working hours, stressful pace, and lack of autonomy)^(7)^.

According to these same authors, in addition to the factors presented above, there are also reports of the presence of violence at school, difficulty in working relationships, little possibility of performing creative activities, lack of time to review homework and tests, and constant political-educational changes. Ferreira et al.^(8)^ carried out a study with teachers from the municipal network of São Paulo and found an association between self-reference to the presence of voice disorder and frequent situations of threat to teachers, aggression, insults, violence outside the school, or against employees.

As for the functional situation, the weekly workload is an aspect frequently addressed in research related to the teacher's voice, as it intensifies the occurrence of vocal symptoms and the emergence of vocal alterations, as reported by Marçal and Peres^(6)^, who found a positive association between vocal disorder and weekly workload.

Considering that several factors can compromise the teacher's vocal production, this study aims to verify whether teachers with less vocal use due to reduced workload have fewer complaints of vocal disorders and better environmental and organizational work conditions.

## METHODS

This is a cross-sectional and descriptive study, approved by the Research Ethics Committee (CAAE 79911817.2.0000.5546, ordinance 2.412.522). All participants signed the Free and Informed Consent Form (FICF), as recommended by regulation 466/2012 of the National Council for Research Ethics.

To compose **group A**, 40 elementary and high school teachers (from 25 to 58 years old) working in public education and linked to a federal public university were invited to participate in the study. All teachers in this group (100%) had a 40 hours/week work routine and exclusive dedication with a minimum workload of 8 and a maximum of 12 hours/week in classrooms with students, according to current legislation. The rest of the workload was divided between research and outreach activities, similar to professors at the Federal Public University. The professors were graduates, with a minimum Ph.D. degree. All permanent teachers at the school were invited to participate in the study, professionals of both genders, with no restrictions on age. Substitutes teachers, visiting scholars, or graduate students who were in teaching internships were excluded from the sample. Those who did not complete the questionnaire or with incorrect answers were also not accepted. Twenty-six teachers acknowledge participating in the study and it was necessary to exclude three individuals due to not filling out the protocols correctly.

**Group B** consisted of 23 elementary and high school teachers from state and municipal public schools, with 52.1%^(9)^ reporting a workload between 11- 20 hours/week, 34.7%^(8)^ a workload of 21-30 hours/week, and 13.9%^(3)^ a workload of 31-40 hours/week of classroom activities with students. The age and gender pairings were performed according to group A. Teachers who were not working in the classroom, with a change of function, and those who did not adequately fill out the protocols were not allowed to participate in the research.

To recruit participants, initially, we contacted the general administration of the federal school (group A) and education secretaries and directors of municipal and state schools (group B). After authorization, the teachers were personally contacted by the researchers, and data collection was scheduled.

All participants answered the Condition of Vocal Production-Teacher questionnaire (CPV-P)^(10)^, consisting of 62 questions that refer to sociodemographic data, functional status, general health aspects, life habits and vocal aspects, and work organization. To differentiate the study groups, the researchers adapted the answer to the following item in the questionnaire: “How many hours a week do you stay with the students?”, instead of the first answer being “one to ten hours”, it was adapted for “one to twelve hours” and, consequently, the other possibilities of answers were also adjusted.

They also responded to the Screening Index for Voice Disorder (SIVD), which integrates the CPV-P and was validated by Ghirardi et al.^(11)^, consisting of 12 vocal symptoms (hoarseness, voice loss, breaking voice, low-pitched voice, phlegm, dry cough, cough with secretion, pain when speaking, pain when swallowing, secretion/phlegm in the throat, dry throat and strained speech) presented in a four-point Likert scale (never, rarely, sometimes and always). For each symptom scored in the “sometimes” or “always” frequencies, one^(1)^ point is computed; the final score is obtained by the sum that can range from zero (0) to 12, and the cutoff point, which constitutes the predictive value of the teacher presenting a probable voice disorder, is ≥5 points.

After establishing the inclusion and exclusion criteria for the groups and acceptance of participation in the research, the final sample consisted of 13 female and 10 male teachers, with a mean age of 39.5 years (±8) old, with a minimum of 25 and up to 57 years old.

Data were tabulated in an Excel^®^ spreadsheet, in which the variables of the CPV-P protocol were selected to perform the descriptive analysis of each group. To compare numerical variables, the Mann-Whitney test was used. The categorical variables were dichotomized; thus, the frequencies “never” and “rarely” were considered as “absent” and “sometimes” and “always” considered as “present”. Fisher's exact test was used for comparative analysis between groups. The accepted significance level was p≤0.05.

## RESULTS

Table 1 presents the comparative results of the numerical variables. Group B had a positive ITDV mean^(5,12)^ for the presence of voice disorder in the sample, and it was possible to observe a statistically significant difference for group A (p=0.02).

**Table 1:** Functional status of teachers: career length and number of schools in which he/she works

Variable	Mean	Standard Deviation (±)	Min	Max	p-value
Career length -Group A	15.4	8.9	25	57	0.86
Career length -Group B	16.35	9.7	2	35.2	
Number of schools in which he/she teaches - Group A	1	0	1	1	<0.0001^*^
Number of schools in which he/she teaches - Group B	2	0.5	1	3	
Voice disorder - Group A	3.04	3.1	0	10	0.02^**^
Voice disorder - Group B	5.21	2.8	0	10	

* Mann Whitney Statistical Test;

** Fisher’s exact test ; p <0.05

Group B had a greater number of hours per week with students (p <0.0001), as Table 2 shows.

**Table 2:** Functional status of teachers: external vocal activities and number of hours with students

Variable	Frequency	Group A		Group B		P-value
		Number	Percentage (%)	Number	Percentage	
External vocal activities	never and rarely	16	69.5	13	56.5	0.27
	sometimes and always	7	30.4	10	43.4	
Number of class hours per week	1 to 10 hours	23	100	0	0	<0.0001^*^
	11 to 20 hours	0	0	12	52.1	
	21 to 30 hours	0	0	8	34.7	
	31 to 40 hours	0	0	3	13.9	

* Fisher's exact test; p <0.05

Aspects of satisfactory acoustics, room temperature, presence of humidity, good lighting, and cleanliness were statistically different between groups, as shown in Table 3. Group B showed greater dissatisfaction with environmental aspects.

**Table 3:** Descriptive and comparative statistics between groups A and B for the work environment

Variable	Parameters	Group A		Group B		p-value
		Number	Percentage (%)	Number	Percentage (%)	
Noisy school	never and rarely	3	13.0	6	26.0	0.23
	sometimes and always	20	86.9	17	73.9	
Satisfactory acoustics	never and rarely	3	13.0	9	39.1	0.04^*^
	sometimes and always	20	86.9	14	60.8	
Dust	never and rarely	11	47.8	10	43.4	0.50
	sometimes and always	12	52.1	13	56.5	
Smoke	never and rarely	23	100	19	86.3	0.10
	sometimes and always	0	0	3	13.6	
Pleasant temperature	never and rarely	3	13.6	9	39.1	0.04*
	sometimes and always	20	86.9	14	60.8	
Humidity	never and rarely	21	91.3	14	60.8	0.01*
	sometimes and always	2	8.7	9	39.1	
Adequate lighting	never and rarely	0	0	9	39.1	0.001*
	sometimes and always	23	100	14	60.8	
School cleanliness	never and rarely	0	0	6	26.0	0.01*
	sometimes and always	23	100	17	73.9	
Adequate classroom size	never and rarely	2	8.7	3	13.0	0.50
	sometimes and always	21	91.3	20	86.9	
Resting place	never and rarely	5	21.7	9	39.1	0.16
	sometimes and always	18	78.2	14	60.8	

* significant p-value if <0.05 – Fischer’s exact test

Table 4 presents the descriptive values as percentages of groups A and B regarding work organization. The variables referring to adequate work material and monotonous work showed a statistically significant difference, which suggests greater dissatisfaction for group B.

**Table 4:** Descriptive and comparative statistics between groups A and B for work organization

Variable	Parameters	Group A		Group B		p-value
		Number	Percentage (%)	Number	Percentage (%)	
Good relationship with co-workers	never and rarely	0	0	0	0	-
	sometimes and always	23	100	23	100	
Good relationship with school management	never and rarely	0	0	0	0	-
	sometimes and always	23	100	23	100	
Good relationship with students	never and rarely	0	0	0	0	-
	sometimes and always	23	100	23	100	
Good relationship with the students’ parents	never and rarely	1	4.3	1	4.3	0.75
	sometimes and always	22	95.6	22	95.6	
Constant supervision	never and rarely	3	13.6	3	13.6	0.63
	sometimes and always	20	86.9	20	86.9	
Stressful work pace	never and rarely	8	34.7	5	21.7	0.25
	sometimes and always	15	65.2	18	78.2	
Appropriate working material	never and rarely	0	0	11	47.8	<0.0001^*^
	sometimes and always	23	100	12	52.1	
Monotone work	never and rarely	20	86.9	12	52.1	0.01*
	sometimes and always	3	13.6	11	47.8	
Time to perform activities at school	never and rarely	2	8.7	7	30.4	0.07
	sometimes and always	21	91.3	16	69.5	
Brings work home	never and rarely	1	4.3	2	8.7	0.50
	sometimes and always	22	95.6	21	91.3	
Job satisfaction	never and rarely	1	4.3	2	8.7	0.50
	sometimes and always	22	95.6	21	91.3	
Stress at work	never and rarely	1	4.3	2	8.7	0.50
	sometimes and always	22	95.6	21	91.3	
Work interferes with health	never and rarely	5	21.7	5	21.7	0.63
	sometimes and always	18	78.2	18	78.2	
Violence Against the teacher	never and rarely	20	86.9	20	86.9	0.30
	sometimes and always	3	13.0	3	13.0	

* significant p-value if <0.05 – Fischer’s test

Table 5 presents the descriptive and comparative values of groups A and B regarding vocal aspects. There is a higher frequency of group B teachers who need to scream (p=0.02) and use their voices in open places (0.02). It is noteworthy that, in group A, 82.6%^(13)^ reported never having received vocal guidance, while in group B 52.1%^(9)^ reported having received guidance on vocal use “sometimes” or “always”.

**Table 5:** Descriptive and comparative statistics between groups A and B for vocal aspects

Variable	Parameters	Group A		Group B		p-value
		Number	Percentage (%)	Number	Percentage (%)	
Screams	never and rarely	11	47.8	4	17.3	0.02*
	sometimes and always	12	52.1	19	82.6	
Speaks a lot	never and rarely	2	8.7	1	4.3	0.5
	sometimes and always	21	91.3	22	95.6	
Speaks in open spaces	never and rarely	10	43.4	3	13.0	0.02*
	sometimes and always	13	56.5	20	86.9	
Preserves voice	never and rarely	3	13.6	5	21.7	0.35
	sometimes and always	20	86.9	18	78.2	
Received vocal guidance	never and rarely	19	82.6	11	47.8	0.01*
	sometimes and always	4	17.3	12	52.1	
Satisfaction with voice	never and rarely	5	21.7	7	30.4	0.36
	sometimes and always	18	78.2	16	69.5	
Calls in sick because of the voice	never and rarely	23	100	22	95.6	0.5
	sometimes and always	0	0	1	4.3	
Smokes	never and rarely	23	100	22	95.6	0.5
	sometimes and always	0	0	1	4.35	
Drinks alcoholic beverages	never and rarely	13	56.5	17	73.9	0.17
	sometimes and always	10	43.4	6	26.0	
Drinks water while using voice	never and rarely	4	17.3	4	17.3	0.65
	sometimes and always	19	82.6	19	82.6	

* significant p-value if <0.05 – Fischer’s test

## DISCUSSION

Characterizing the risks present in the school is appropriate, as it allows planning and developing actions that favor a healthy environment to live in and that promote the quality of life of all segments that work there, especially teachers.

This research instrument is used in several studies to know the working conditions of teachers. The multifactorial characteristic of the teachers' work environment can be a risk factor for the development of voice disorders, impacting professional performance^(14)^.

It is common to observe in studies the high vocal demand for teachers due to excessive workload, low pay, and the need to work in several schools to complement the family income. The idea of conducting this research occurred at the beginning of outreach activities in a school linked to the Federal Public University, in which it was observed that the teachers had an exclusive dedication regime, remuneration similar to that of a federal graduate teacher, maximum classroom workload of 12 hours/class, the possibility of developing research and extension activities, and assistance for academic scholarships and internships. These working conditions are rarely observed in the Brazilian public education system.

Thus, it was questioned whether, in addition to these, there would be other differences related to work situations that could be linked to the environment, work organization, and vocal aspects.

In the present study, the mean age of the group of teachers was 39.5 years old (SD=8), similar to other works on the subject^(9,15,16)^. The career length in the profession was similar between the two groups, 15 years for group A and 16 years for group B, similar to a study carried out with 272 teachers in the city of São Paulo^(17)^.

The analysis of the functional situation is a factor that interferes with their vocal production condition, that is, the time (hours) that the teacher remains in the classroom and the number of schools he/she teaches may interfere with vocal quality, which was an important difference observed between the two groups studied in this research.

In the present study, it was observed that teachers in group B work in an average of two to three schools, as they do not have exclusive dedication. Thus, they spend more weekly hours in the classroom due to low pay and the need to work in several schools to be able to increase their income. This data was also evidenced in the study with teachers from the private network of Bahia^(18)^ and from the state education network in the cities of Campinas and São José do Rio Pardo^(19)^, demonstrating that in the state, municipal, and private education systems these professionals are underpaid.

Regarding factors related to the work environment (Table 3), it was observed that teachers in group A report better acoustic (p=0.04), temperature (p=0.04), humidity (p=0 .01), lighting (p=0.001), and cleaning (p=0.01) situations. Satisfaction with school cleanliness can be explained by the financial resources allocated to hiring professionals to perform this function in these schools. The unsatisfactory cleanliness of the school was also reported in a study carried out by Ferreira et al.^(20)^ with teachers from the municipal network of São Paulo.

The other variables related to the work environment (noisy school, satisfactory acoustics, presence of dust, smoke, humidity, pleasant temperature, adequate lighting, adequate classroom size, and resting place) did not show a significant difference between the groups. However, via the descriptive analysis in percentage values, some characteristics that deserve to be highlighted among the populations studied were observed.

The presence of noise was frequent in both groups (A-86.96% and B-73.91%). The presence of noise in the school environment had similar results in other studies^(13,21)^. Fortes et al.^(12)^ emphasize that the main factor for the emergence of a voice disorder is its intensive use related to harmful environmental factors, such as exposure to noise. Mendes et al.^(22)^ inferred that inadequate working conditions, with constant exposure to noise at high levels, increased the intensity of the voice of teachers and, consequently, an overload on the vocal tract, with a reduction in the extension of the vocal tract, predisposing to the development of auditory and sensory vocal symptoms or even signaling voice disorders.

Unsatisfactory acoustics were most mentioned by teachers in group B (39.13%). This information brings an important reflection on the structural aspects of these schools. The unsatisfactory acoustic reflects directly on vocal production, as there will be a need for the individual to increase the intensity of their voice so that all students can obtain auditory information. Under unfavorable acoustic conditions, the student will present difficulties in understanding the message^(23)^, which impairs the teaching and learning process and can also create stress for the teacher^(23)^.

Teachers in this same group also had greater complaints about unpleasant temperatures (39.13%). During the research, it was observed that the classrooms of group A all had air conditioning, unlike what was seen in the other group, in which ventilation was carried out by ventilators that were not always in good condition. The hot climate of the region where the study was carried out justifies the need for an adequate temperature for the development of teaching activities, as well as for student learning. Heat is considered one of the greatest environmental stressors. The lack of thermal comfort is responsible for the main complaints of education workers, mainly teachers^(23)^.

Good lighting can make the classroom more pleasant, providing comfort, little fatigue, and little monotony, which contribute to improving the performance of people present in the environment. Teachers in group B reported inadequate lighting conditions (39.13%). Inadequate lighting in the classroom can damage the visual health of people in the environment and worsen conditions for those with vision problems. It can also trigger fatigue, headache, and eye irritability processes, and directly interfere in the performance of the teacher and students^(23)^.

Teachers in group B had a greater complaint of monotonous work and dissatisfaction with work materials. This leads us to think once again about the financial incentives in education for the purchase of support materials and possibilities to diversify the work of teachers.

The habit of screaming was observed with a high percentage in both groups, being worse in group B (p=0.02). Studies show that screaming interferes with healthy voice production and the occurrence of voice disorder may be related to these habits^(24)^. Environmental factors such as noise, unfavorable acoustics, and the need to work outdoors, which were observed with greater prevalence in this group, make teachers look for this compensatory strategy (screaming) that can cause the presence of vocal symptoms such as hoarseness, vocal fatigue, and sore throat.

Group A reported receiving less vocal guidance (p=0.01), perhaps because many teachers only seek guidance when vocal symptoms appear^(2)^. Another possible relationship is the lack of vocal health promotion actions aimed at this group of teachers in the analyzed school. Obtaining this information helps in better voice care and self-knowledge, reducing vocal complaints. It is important to emphasize that the participating teachers received vocal guidance after data collection and analysis, in addition to feedback on their vocal situation and necessary guidance.

The perception of teachers about their voice, specifically regarding the identification of alterations present in them, has been a recurrent theme in Speech, Language and Hearing Sciences research. The present study corroborates the literature regarding the presence of voice disorder in teachers^(21,25)^. However, only teachers with a workload of more than 20 hours (group B) had a positive mean for the presence of voice disorder.

The higher prevalence of voice disorder in this group of teachers can be explained by the workload, as well as by the vocal production condition factors (environment, work organization, and vocal habits).

The consequences of voice disorder for the teacher go beyond the vocal problem, causing negative interference in the performance of their work and difficulties in relationships with peers and presenting social, economic, professional, and personal impacts, which can lead to permanent professional leave^(26)^.

The identification of factors that interfere with vocal production is of paramount importance since, when detected, it is possible to plan actions to prevent vocal disorders and promote vocal health.

It is noteworthy that this study was not the object of investigation to verify which sphere of education has a better condition of vocal production, but rather to analyze whether teachers with reduced hours, less time in the classroom, and linked to only one school have better working conditions and complaint less of voice disorder. Perhaps, group B had the biggest complaints due to their workload.

The two contexts analyzed showed significant differences at the functional (teachers' workload in the classroom and number of schools they teach), organizational (adequate work material and monotonous work), and work environment (satisfactory acoustics, pleasant temperature, adequate lighting, presence of humidity and cleanliness of the school) levels.

Thus, it is possible to reflect on the possibility that the working conditions of public teaching schools related to organizational and environmental aspects are similar and are rooted in school contexts. Both groups report high work demand, stress, the presence of work outside the school, and work interference in health, as seen in studies carried out with teachers in Brazil^(17)^.

Another prominent factor observed in this study, which differs from other research carried out in Brazilian public schools, corresponds to the low situation of violence in schools and the satisfaction of teachers in the performance of their professional functions. Working experience in a socially “appropriate” environment allows teachers to perform their duties satisfactorily, contributing positively to student learning.

Regardless of the type of public school, it is important to understand the educational process, relating the functional, organic, emotional, and social aspects so that speech therapy work is effective and thus able to promote vocal well-being. The factors pointed out in this study should be considered in the formulation and implementation of preventive measures for the vocal health of teachers.

The differentiation of groups by workload seemed to demonstrate an important factor for the teacher's vocal production condition. However, in the present study, an analysis was carried out based on the teacher's self-report. Dosing the time of voice use within this workload reported by the teachers and relating it to the CPV-P variables may be a theme for a future study.

## CONCLUSION

Teachers from the school system belonging to the group of exclusive dedication to a single school, with reduced weekly workload in the classroom and less vocal exposure had fewer complaints of voice disorders, better environmental and organizational conditions, and reported screaming less in the classroom workplace.
