# Supplementary material for: Vocal teacher production condition in differents functional situations
Source: Codas. 2021 Dec 8;34(1):e20200208. doi: 10.1590/2317-1782/20202020208 (PMC9886111; doi:10.1590/2317-1782/20202020208)
Supplement: Translation

# Translation: Condição de produção vocal do professor em diferentes situações funcionais

**Authors:** Aline Ferreira de Brito Mota, Ariane Damasceno Pellicani, Rodrigo Dornelas, Lilian Neto Aguiar Ricz

**Affiliations:** 1 Departamento de Fonoaudiologia, Universidade Federal de Sergipe – UFS - Lagarto (SE), Brasil.; 2 Programa de Pós-graduação do Departamento de Otorrinolaringologia, Oftalmoologia e Cabeça e Pescoço, Faculdade de Medicina de Ribeirão Preto – FMRP, Universidade de São Paulo – USP - Ribeirão Preto (SP), Brasil.; 3 Departamento de Fonoaudiologia, Universidade Federal do Rio de Janeiro – UFRJ - Rio de Janeiro (RJ), Brasil.; 4 Departamento de Oftalmologia, Otorrinolaringologia e Cirurgia de Cabeça e Pescoço, Faculdade de Medicina de Ribeirão Preto – FMRP, Universidade de São Paulo – USP - Ribeirão Preto (SP), Brasil.

**Keywords:** Docentes, Disfonia, Educação, Voz, Fonoaudiologia

## Abstract

**Objetivo:**

verificar se professores com menor uso vocal em decorrência da carga horária de trabalho reduzida apresentam menor queixa de distúrbio vocal e melhores condições ambientais e organizacionais de trabalho.

**Método:**

participaram do estudo 46 professores, de ambos os gêneros, com média de idade de 39,5 anos (DP=8), tempo de profissão médio de 15 anos. Os professores foram divididos em: grupo A aqueles com regime de dedicação exclusiva, vinculados a uma única escola pública de ensino e carga horária de sala de aula reduzida; grupo B, professores da rede pública de ensino que trabalhavam em várias escolas e com hora aula elevada. Todos os sujeitos foram submetidos aos seguintes instrumentos: Condição de Produção Vocal – Professor (CPV-P) e o Índice de Triagem para Distúrbio de Voz (ITDV).

**Resultados:**

o grupo B apresentou presença de distúrbio de voz (5,21; p=0,02) e maiores queixas quanto a condições de acústica (p=0,04), temperatura (p=0,04), umidade (p=0,01), iluminação (p=0,001), limpeza (p=0,01), material didático (p<0,0001). Hábitos de gritar (p=0,02), falar em local aberto (p=0,02) e receber orientações vocais (p=0,01) também apresentaram diferença estatisticamente significantes.

**Conclusão:**

Os professores atuantes no ensino fundamental e médio de ensino pertencentes ao grupo de vínculo de dedicação exclusiva a uma única escola, com hora semanal reduzida de sala de aula e menor exposição vocal apresentaram menor queixa de distúrbio de voz, melhores condições ambientais e organizacionais e referiram gritar menos no ambiente de trabalho.

## INTRODUÇÃO

Os professores fazem parte dos profissionais que têm na comunicação elemento vital para a viabilização de seu trabalho, sendo a voz o instrumento utilizado para estabelecer vínculos diretos com o aluno, a família e a comunidade^(1,2)^.

Distúrbios vocais podem ser mais frequentes entre a população que necessita utilizar a voz profissionalmente, devido à grande demanda vocal e exposição aos diversos fatores de risco. Voz profissional foi conceituada como uma forma de comunicação oral, utilizada por indivíduos que dela dependem para exercer sua atividade ocupacional e, por meio desse modo de expressão, atingir um público específico e determinado^(3,4)^.

Os professores representam a categoria de profissionais mais afetada pelo distúrbio de voz^(5)^. O distúrbio de voz é definido como qualquer alteração da voz decorrente de um distúrbio funcional e/ou orgânico do trato vocal que impossibilite a produção natural da voz^(6)^.

Na busca para o tratamento do distúrbio de voz, os professores relatam que o adoecimento possui estreita relação com o ambiente e a organização do trabalho. A associação das condições do trabalho docente ao prejuízo da produção da voz é ressaltada em relação aos aspectos ambientais da escola (como o ruído que obriga o uso da voz em intensidade mais elevada, ou a poeira que desencadeia reações alérgicas), quanto à organização do trabalho docente (como jornadas prolongadas de trabalho, ritmo estressante e falta de autonomia)^(7)^.

Segundo esses mesmos autores, além dos fatores apresentados anteriormente, existem relatos também da presença de violência na escola, dificuldade de relacionamento no trabalho, pouca possibilidade de criatividade nas atividades, falta de tempo para correção de tarefas e provas, e constantes mudanças político-educacionais. Ferreira et al. ^(8)^ realizaram um estudo com professores da rede municipal de São Paulo e constataram a associação entre autorreferência à presença de distúrbio de voz e situações frequentes de ameaça ao professor, agressões, insultos, violência à porta da escola ou contra os funcionários.

Quanto à situação funcional, a carga horária semanal é um aspecto frequentemente abordado nas pesquisas relacionadas à voz do professor, por intensificar a ocorrência de sintomas vocais e o surgimento de alterações vocais como é relatado por Marçal e Peres^(6)^, que encontraram associação positiva entre distúrbio vocal e carga horária semanal.

Tendo em vista que diversos fatores podem vir a comprometer a produção vocal do professor, este estudo tem como objetivo verificar se professores com menor uso vocal em decorrência da carga horária de trabalho reduzida apresentam menor queixa de distúrbio vocal e melhores condições ambientais e organizacionais de trabalho.

## MÉTODO

Trata-se de um estudo transversal, descritivo, aprovado pelo Comitê de Ética em Pesquisa (CAAE 79911817.2.0000.5546, parecer 2.412.522). No qual todos os participantes assinaram o Termo de Consentimento Livre e Esclarecido (TCLE), conforme a recomendação da norma 466/2012 da Conselho Nacional de Ética em Pesquisa.

Para compor o **grupo A** foram convidados a participar do estudo os 40 professores do ensino fundamental e médio (de 25 a 58 anos) atuantes no ensino público vinculado a uma universidade pública federal. Todos os professores deste grupo (100%) tinham jornada de trabalho de 40h/semanais e vínculo de dedicação exclusiva com carga horária mínima de 8 e máxima de 12h/ semana de sala de aula com os alunos, conforme legislação vigente. O restante da carga horária era dividido entre atividades de pesquisa e extensão, semelhante a docentes da Universidade Pública Federal. Os professores eram pós-graduados, com titulação mínima de Doutorado. Todos os professores efetivos da escola foram convidados a participar do estudo, profissionais de ambos os gêneros, sem restrições quanto à idade. Foram excluídos da amostra professores com vínculo de substitutos, convidados ou pós-graduandos que estivessem em estágio docente. Também não foram aceitos aqueles que não preencheram o questionário na íntegra ou com respostas incorretas. Aceitaram participar do estudo 26 professores e foi necessária a exclusão de três indivíduos devido ao não preenchimento correto dos protocolos.

O **grupo B** foi composto por 23 professores do ensino fundamental e médio de escola pública estadual e municipal, sendo que 52,1%^(9)^ relataram carga horária entre 11 2 20horas/semanais, 34,7%^(8)^ carga horária de 21-30 horas/semanais e 13,9%^(3)^, carga horária de 31-40 horas/semanais de sala de aula com os alunos. Foi realizado o pareamento de idade e gênero de acordo com o grupo A. Não foram aceitos a participar da pesquisa os professores que não estivessem atuando na sala de aula, com mudança de função e aqueles que não preenchessem adequadamente os protocolos.

Para o recrutamento dos participantes, inicialmente, foi realizado um contato com a administração geral da escola federal (grupo A) e secretários de educação e direções das escolas municipais e estaduais (grupo B). Após a autorização, os professores foram contactados pessoalmente pelos pesquisadores e agendada a coleta de dados.

Todos os participantes responderam ao questionário *Condição de produção vocal do professor CPV-P* ^(10)^ composto por 62 questões que fazem referência a dados sociodemográficos, situação funcional, aspectos gerais de saúde, hábitos de vida e aspectos vocais e organização do trabalho. De forma a diferenciar os grupos de estudo, os pesquisadores realizaram uma adaptação na forma de resposta da seguinte pergunta do questionário: “Quantas horas por semana você permanece com os alunos? ”, ao invés da primeira resposta ser de “uma a dez horas”, foi adaptado para “uma a doze horas” e, consequentemente, ajustadas as outras possibilidades de respostas.

Também responderam ao Índice de triagem de distúrbio de voz *(ITDV)*, que integra o CPV-P e foi validado por Ghirardi et al.^(11)^, composto por 12 sintomas vocais (rouquidão, perda da voz, falha na voz, voz grossa, pigarro, tosse seca, tosse com secreção, dor ao falar, dor ao engolir, secreção na garganta, garganta seca e cansaço ao falar) apresentados em escala *Likert* de quatro pontos (nunca, raramente, às vezes e sempre). A cada sintoma assinalado nas frequências às vezes ou sempre, um^(1)^ ponto é computado; o escore final é obtido pela somatória que pode variar de zero (0) a 12, e o ponto de corte, que se constitui no valor preditivo do professor apresentar um provável distúrbio de voz é ≥5 pontos.

Após o estabelecimento dos critérios de inclusão e exclusão referentes ao grupos e aceitação da participação na pesquisa, a amostra final foi composta por 13 professores do gênero feminino e 10 masculino, com idade média de 39,5 anos (±8), com mínima de 25 e máximo de 57 anos.

Os dados foram tabulados em planilha *Excel*®, no qual foram selecionadas as variáveis do protocolo CPV-P para realizar a análise descritiva de cada grupo. Para comparar as variáveis numéricas foi utilizado o teste Mann-Whitney. As variáveis categóricas foram dicotomizadas, assim, as frequências nunca e raramente foram consideradas como “ausente” e as vezes e sempre, consideradas com “presente”. O teste exato de *Fisher* foi utilizado para a análise comparativa entre os grupos. O nível de significância aceito foi p≤0,05.

## RESULTADOS

A Tabela 1 apresenta os resultados comparativos das variáveis numéricas. O grupo B apresentou média do ITDV positiva^(5,12)^ para a presença do distúrbio de voz na amostra, sendo possível observar diferença estatisticamente significante para o grupo A (p=0,02).

**Tabela 1:** Situação funcional dos professores: tempo de profissão e número de escolas lecionadas

Variável	Média	Desvio Padrã0 (±)	Min	Max	p-valor
Tempo de profissão -Grupo A	15,4	8,9	25	57	0,86
Tempo de profissão -Grupo B	16,35	9,7	2	35,2	
Nº de escolas que leciona - Grupo A	1	0	1	1	<0,0001*
Nº de escolas que leciona- Grupo B	2	0,5	1	3	
Distúrbio de Voz - Grupo A	3,04	3,1	0	10	0,02**
Distúrbio de Voz - Grupo B	5,21	2,8	0	10	

* Teste estatístico de Mann Whitney

** Teste exato de Fisher ; p <0,05

O grupo B apresentou maior quantidade de horas/ semana com o aluno (p <0,0001), como é possível observar na Tabela 2.

**Tabela 2:** Situação funcional dos professores: atividades vocais externas e quantidade de horas com alunos

Variável	Frequência	Grupo A		Grupo B		P- valor
		Nº Absoluto	Porcentagem (%)	Nº Absoluto	Porcentagem (%)	
Atividades vocais externas	nunca e raramente	16	69,5	13	56,5	0,27
	as vezes e sempre	7	30,4	10	43,4	
Quantidade de horas-aula por semana	1 a 10 horas	23	100	0	0	<0,0001*
	11h a 20 horas	0	0	12	52,1	
	21 a 30 horas	0	0	8	34,7	
	31h a mais de 40 horas	0	0	3	13,9	

* Teste exato de Fisher ; p <0,05

Os aspectos de acústica satisfatória, temperatura da sala, presença de umidade, boa iluminação e limpeza apresentaram-se diferentes estatisticamente entre os grupos, como demonstrado na Tabela 3. O grupo B apresentou maior insatisfação com relação aos aspectos ambientais.

**Tabela 3:** Estatística descritiva e comparativa entre os grupos A e B para o ambiente de trabalho

Variável	Parâmetros	Grupo A		Grupo B		p-valor
		Nº Absoluto	Porcentagem (%)	Nº Absoluto	Porcentagem (%)	
Ruído na escola	nunca e raramente	3	13,0	6	26,0	0,23
	as vezes e sempre	20	86,9	17	73,9	
Acústica Satisfatória	nunca e raramente	3	13,0	9	39,1	0,04*
	as vezes e sempre	20	86,9	14	60,8	
Poeira	nunca e raramente	11	47,8	10	43,4	0,50
	as vezes e sempre	12	52,1	13	56,5	
Fumaça	nunca e raramente	23	100	19	86,3	0,10
	as vezes e sempre	0	0	3	13,6	
Temperatura agradável	nunca e raramente	3	13,6	9	39,1	0,04*
	as vezes e sempre	20	86,9	14	60,8	
Presença de umidade	nunca e raramente	21	91,3	14	60,8	0,01*
	as vezes e sempre	2	8,7	9	39,1	
Iluminação adequada	nunca e raramente	0	0	9	39,1	0,001*
	as vezes e sempre	23	100	14	60,8	
Limpeza da escola	nunca e raramente	0	0	6	26,0	0,01*
	as vezes e sempre	23	100	17	73,9	
Tamanho da sala adequado	nunca e raramente	2	8,7	3	13,0	0,50
	as vezes e sempre	21	91,3	20	86,9	
Local de descanso	nunca e raramente	5	21,7	9	39,1	0,16
	as vezes e sempre	18	78,2	14	60,8	

* p-valor <0,05 considerado significante – teste de Fischer

A Tabela 4 apresenta os valores descritivo em porcentagem do grupo A e do grupo B que diz respeito a organização de trabalho. As variáveis referentes ao material de trabalho adequado e trabalho monótono apresentaram diferença estatisticamente significante, o que sugere maior insatisfação para o grupo B.

**Tabela 4:** Estatística descritiva e comparativa entre os grupos A e B para a organização de trabalho

Variável	Parâmetros	Grupo A		Grupo B		p-valor
		Nº Absoluto	Porcentagem (%)	Nº Absoluto	Porcentagem (%)	
Bom relacionamento com colegas de trabalho	nunca e raramente	0	0	0	0	-
	as vezes e sempre	23	100	23	100	
Bom relacionamento com direção da escola	nunca e raramente	0	0	0	0	-
	as vezes e sempre	23	100	23	100	
Bom relacionamento com alunos	nunca e raramente	0	0	0	0	-
	as vezes e sempre	23	100	23	100	
Bom relacionamento com pais de alunos	nunca e raramente	1	4,3	1	4,3	0,75
	as vezes e sempre	22	95,6	22	95,6	
Supervisão constante	nunca e raramente	3	13,6	3	13,6	0,63
	as vezes e sempre	20	86,9	20	86,9	
Ritmo de trabalho estressante	nunca e raramente	8	34,7	5	21,7	0,25
	as vezes e sempre	15	65,2	18	78,2	
Material de trabalho adequado	nunca e raramente	0	0	11	47,8	<0,0001*
	as vezes e sempre	23	100	12	52,1	
Trabalho monótono	nunca e raramente	20	86,9	12	52,1	0,01*
	as vezes e sempre	3	13,6	11	47,8	
Tempo para realizar atividades na escola	nunca e raramente	2	8,7	7	30,4	0,07
	as vezes e sempre	21	91,3	16	69,5	
Leva trabalho para casa	nunca e raramente	1	4,3	2	8,7	0,50
	as vezes e sempre	22	95,6	21	91,3	
Satisfação na função	nunca e raramente	1	4,3	2	8,7	0,50
	as vezes e sempre	22	95,6	21	91,3	
Estresse no trabalho	nunca e raramente	1	4,3	2	8,7	0,50
	as vezes e sempre	22	95,6	21	91,3	
Trabalho interfere na saúde	nunca e raramente	5	21,7	5	21,7	0,63
	as vezes e sempre	18	78,2	18	78,2	
Violência contra professor	nunca e raramente	20	86,9	20	86,9	0,30
	as vezes e sempre	3	13,0	3	13,0	

* p-valor <0,05 considerado significante – teste de Fischer

A Tabela 5 apresenta os valores descritivo e comparativo do grupo A e do grupo B que diz respeito aos aspectos vocais. É maior a frequência de professores do grupo B que necessitam gritar (p=0,02) e utilizar a voz em locais abertos (0,02). Destaca-se que no grupo A 82,6%^(13)^ relataram nunca terem recebido orientações vocais, enquanto que no grupo B 52,1%^(9)^ relataram terem recebido orientações de uso vocal as vezes ou sempre.

**Tabela 5:** Estatística descritiva e comparativa entre os grupos A e B para a aspectos vocais

Variável	Parâmetros	Grupo A		Grupo B		p-valor
		Nº Absoluto	Porcentagem (%)	Nº Absoluto	Porcentagem (%)	
Grita	nunca e raramente	11	47,8	4	17,3	0,02*
	as vezes e sempre	12	52,1	19	82,6	
Fala muito	nunca e raramente	2	8,7	1	4,3	0,5
	as vezes e sempre	21	91,3	22	95,6	
Fala em lugar aberto	nunca e raramente	10	43,4	3	13,0	0,02*
	as vezes e sempre	13	56,5	20	86,9	
Poupa a voz	nunca e raramente	3	13,6	5	21,7	0,35
	as vezes e sempre	20	86,9	18	78,2	
Recebeu orientações vocais	nunca e raramente	19	82,6	11	47,8	0,01*
	as vezes e sempre	4	17,3	12	52,1	
Satisfeito com a voz	nunca e raramente	5	21,7	7	30,4	0,36
	as vezes e sempre	18	78,2	16	69,5	
Falta no trabalho por causa da voz	nunca e raramente	23	100	22	95,6	0,5
	as vezes e sempre	0	0	1	4,3	
fuma	nunca e raramente	23	100	22	95,6	0,5
	as vezes e sempre	0	0	1	4,35	
Uso de bebida alcoólica	nunca e raramente	13	56,5	17	73,9	0,17
	as vezes e sempre	10	43,4	6	26,0	
Bebe água durante uso da voz	nunca e raramente	4	17,3	4	17,3	0,65
	as vezes e sempre	19	82,6	19	82,6	

* p-valor <0,05 considerado significante – teste de Fischer

## DISCUSSÃO

Caracterizar os riscos presentes na escola é apropriado, uma vez que permite planejar e desenvolver ações que favoreçam um ambiente saudável para se viver e que promovam a qualidade de vida de todos os segmentos que lá trabalham, em especial o professor.

O instrumento usado nessa pesquisa é utilizado em diversos estudos com a finalidade de conhecer as condições de trabalho do professor. A característica multifatorial do ambiente de trabalho dos professores pode ser um fator de risco para o desenvolvimento de distúrbios de voz, ocasionando impacto no desempenho profissional^(14)^.

É frequente observar em estudos a alta demanda vocal por professores devido a carga horária excessiva, com baixa remuneração e necessidade de trabalhar em várias escolas para complementar a renda familiar. A ideia de realizar esta pesquisa ocorreu no início de uma atividade de extensão na escola vinculada a Universidade Pública Federal, no que foi observado que os professores apresentavam regime de dedicação exclusiva, remuneração semelhante ao de um professor de graduação federal, carga horária máxima de sala de 12 horas/aula e possibilidade de desenvolvimento de pesquisas e atividades de extensão, auxílio de bolsistas acadêmicos e estagiários. Estas condições de trabalho são raramente observadas na rede pública de ensino brasileira.

Desta forma, questionou-se se além destas haveriam outras diferenças relacionadas a situações de trabalho que poderiam estar vinculadas ao ambiente, organização de trabalho e aspectos vocais.

No presente estudo, a média de idade do grupo de professores foi de 39,5 anos (DP=8) similar à de outras pesquisas com docentes^(9,15,16)^. O tempo de profissão foi semelhante entre os dois grupos, 15 anos para o grupo A e 16 anos para o grupo B como também encontrado em um estudo realizado com 272 docentes da cidade de São Paulo^(17)^.

A análise da situação funcional é um fator que interfere na sua condição de produção vocal, ou seja, o tempo (horas) que o professor permanece em sala de aula e a quantidade de escolas que leciona poderá interferir na qualidade vocal, que foi uma importante diferença observada entre os dois grupos estudados nesta pesquisa.

No presente estudo foi observado que os professores do grupo B trabalham em média de duas a três escolas, visto que não apresentam dedicação exclusiva. Assim, permanecem mais horas semanais em sala de aula devido à baixa remuneração e necessidade de trabalhar em várias escolas para conseguir aumentar sua renda. Esse dado também foi evidenciado no estudo com professores da rede particular da Bahia^(18)^ e da rede estadual de ensino nas cidades de Campinas e São José do Rio Pardo^(19)^, demonstrando que tanto na rede estadual, municipal e particular de ensino ocorre uma baixa remuneração desses profissionais.

No que diz respeito aos fatores relativos ao ambiente de trabalho (Tabela 3), foi observado que professores do grupo A relatam melhores situações de acústica (p=0,04), temperatura (p=0,04), umidade (p=0,01), iluminação (p=0,001) e limpeza (p=0,01). A satisfação com a limpeza da escola pode ser explicada pelos recursos financeiros destinados a contratação de profissionais para o desempenho dessa função nessas escolas. A limpeza insatisfatória da escola também foi referida em um estudo realizado por Ferreira et al.^(20)^ com professores da rede municipal de São Paulo.

As demais variáveis relativas ao ambiente de trabalho (escola ruidosa, acústica satisfatória, presença de poeira, fumaça, umidade, temperatura agradável, iluminação adequada, tamanho adequado da sala de aula e local para descanso) não evidenciaram uma diferença significativa entre os grupos. Mas por meio da análise descritiva em valores de porcentagem observou-se algumas características que merecem ser destacadas entre as populações estudadas.

A presença de ruído foi frequente nos dois grupos estudados (A-86,96‬% e B-73,91%). A presença de ruído em ambiente escolar teve resultado semelhante em outras pesquisas^(13,21)^. Fortes et al.^(12)^ destacam que o principal fator para o surgimento de um distúrbio da voz é o uso intensivo da voz relacionado a fatores ambientais prejudiciais, como a exposição ao ruído. Mendes et al.^(22)^ inferiu que condições de trabalho inadequadas, com constante exposição a ruídos em níveis elevados gerou elevação da intensidade da voz de professoras e, consequentemente, sobrecarga no aparelho fonador, com diminuição da extensão do trato vocal, predispondo ao desenvolvimento de sintomas vocais auditivos e sensoriais ou mesmo sinalizando quadros de distúrbios da voz.

A acústica insatisfatória foi mais referida pelos professores do grupo B (39,13‬%). Tal dado traz uma reflexão importante quanto aos aspectos estruturais dessas escolas. A acústica insatisfatória reflete diretamente na produção vocal, pois haverá a necessidade de o indivíduo aumentar a intensidade da sua voz para que todos os discentes possam obter a informação via auditiva. Em condições de acústica desfavorável, o aluno terá dificuldade em compreender a mensagem^(23)^ o que acarreta prejuízo no processo de ensino e aprendizagem e, além disso, pode ainda gerar estresse ao professor^(23)^.

Os professores desse mesmo grupo também apresentaram maiores queixas quanto a temperatura desagradável (39,13%). Durante a realização da pesquisa observou-se que as salas de aulas do grupo A todas possuíam ar-condicionado, diferentemente do que foi visto no outro grupo, em que a ventilação era realizada por aparelhos ventiladores nem sempre em boas condições de uso. O clima quente da região que o estudo foi realizado justifica a necessidade de uma temperatura adequada para desenvolvimento das atividades de docência, como também para a aprendizagem dos alunos. O calor é considerado um dos grandes estressores ambientais. A falta de conforto térmico é responsável pelas principais reclamações dos trabalhadores da educação, especificamente, os professores^(23)^.

Uma boa iluminação pode tornar mais agradável a sala de aula, proporcionando conforto, pouca fadiga e pouca monotonia, podendo contribuir com a melhora do desempenho das pessoas presentes no ambiente. Os professores do grupo B referiram condições de iluminação inadequadas (39,13%). Uma iluminação inadequada na sala de aula pode acarretar danos à saúde visual das pessoas no ambiente e uma piora para os que apresentam problemas de visão. Pode também desencadear processos de fadiga, cefaleia e irritabilidade ocular, e interferir diretamente no desempenho do professor e dos alunos^(23)^.

Os professores do grupo B apresentaram maior queixa de trabalho monótono e insatisfação com os materiais de trabalho. O que nos leva a refletir mais uma vez quanto aos incentivos financeiros na educação para a compra de materiais de apoio e possibilidades de diversificar o trabalho dos professores.

O hábito de gritar foi observado com alta porcentagem em ambos os grupos, sendo pior para o grupo B (p=0,02). Estudos apontam que o grito interfere na produção saudável da voz e a ocorrência do distúrbio de voz pode estar relacionado a esses hábitos^(24)^. Os fatores ambientais como ruído, acústica desfavorável e necessidade de trabalhar em lugar aberto, que foram observados com maior prevalência nesse grupo, faz com que os professores busquem essa estratégia compensatória (grito) que podem vir a ocasionar a presença de sintomas vocais como rouquidão, fadiga vocal e dor na garganta.

O grupo A foi o que menos referiu ter recebido orientações vocais (p=0,01), talvez devido ao fato de muitos professores procurarem orientações apenas quando surgem os sintomas vocais^(2)^. Outra possível relação é por não ocorrer ações de promoção de saúde vocal destinados a esse grupo de professores na escola analisada. A obtenção dessas informações auxilia no melhor cuidado com a voz e autoconhecimento reduzindo as queixas vocais. É importante ressaltar que os professores participantes receberam orientações vocais após a coleta e análise dos dados, além do *feedback* de sua situação vocal e orientações necessárias.

A percepção de professores sobre sua própria voz, especificamente quanto à identificação de alterações nelas presentes, tem sido um tema recorrente nas pesquisas fonoaudiológicas. O presente estudo corrobora a literatura quanto a presença de distúrbio de voz em professores^(21,25)^. No entanto, apenas professores com carga horária acima de 20 horas (grupo B) apresentaram média positiva para a presença do distúrbio de voz.

A maior prevalência do distúrbio de voz nesse grupo de professores pode ser justificada pela carga horária, assim como pelos fatores de condição de produção vocal (ambiente, organização de trabalho e hábitos vocais).

As consequências do distúrbio de voz para o professor vão além do problema vocal, ocasiona interferências negativas no desempenho de seu trabalho e dificuldades de relacionamento com os pares, impactos de ordem social, econômica, profissional e pessoal, podendo ocasionar o afastamento funcional definitivo^(26)^.

O reconhecimento dos fatores que interferem na produção vocal é de suma importância, uma vez que, quando detectados, é possível planejar ações de prevenção de distúrbios vocais e a promoção da saúde vocal.

Ressalta-se que não foi objeto de investigação deste estudo verificar qual esfera de ensino apresenta melhor condição de produção vocal, mas sim analisar se professores com carga horária reduzida, menor tempo de sala de aula e vínculo a apenas uma escola apresenta melhores condições de trabalho e menor queixa de distúrbio de voz. Talvez, o grupo B tenha apresentado maiores queixas devido a sua carga horária de trabalho.

Os dois contextos analisados apresentaram diferenças significantes a nível funcional (carga horária dos professores em sala de aula e quantidade de escolas que lecionam), organizacional (material de trabalho adequado e trabalho monótono) e ambiente de trabalho (acústica satisfatória, temperatura agradável, iluminação adequada, presença de umidade e limpeza da escola).

Assim, é possível refletir na possibilidade de as condições de trabalho das escolas públicas de ensino relacionados aos aspectos organizacionais e ambientais serem semelhantes e estarem enraizadas nos contextos escolares. Os dois grupos relatam alta demanda de trabalho, estresse, presença de trabalho para além da escola e interferência do trabalho na saúde, como é visto em estudos realizados com professores no Brasil^(17)^.

Outro fator de destaque observado nesse estudo que difere de outras pesquisas realizadas em escolas públicas brasileiras, corresponde a baixa situação de violência nas escolas e a satisfação dos professores no desempenho de suas funções profissionais. A vivência laboral em um ambiente socialmente “adequado” permite que os professores desempenhem suas funções de forma satisfatória, contribuindo de forma positiva para a aprendizagem dos alunos.

Independentemente do tipo de escola pública, é importante que se compreenda o processo educacional, relacionando os aspectos funcionais, orgânicos, emocionais e sociais para que a atuação fonoaudiológica seja efetiva e assim consiga promover o bem-estar vocal. Os fatores apontados neste estudo devem ser considerados na formulação e na execução de medidas preventivas a saúde vocal de professores.

A diferenciação dos grupos por meio da carga horária pareceu demonstrar um fator importante para a condição de produção vocal do professor. Entretanto, no presente estudo foi uma análise realizada em função do autorrelato do professor. Dosar o tempo de uso de voz dentro dessa carga horária relatada pelos professores e relacionar com as variáveis do CPV-p pode ser um tema para um próximo estudo.

## CONCLUSÃO

Os professores da rede de ensino pertencentes ao grupo de vínculo de dedicação exclusiva a uma única escola, com carga horária semanal reduzida de sala de aula e menor exposição vocal apresentaram menor queixa de distúrbio de voz, melhores condições ambientais e organizacionais e referiram gritar menos no ambiente de trabalho.
